# Auto-immune skin diseases in animals: time to reclassify and review after 40 years

**DOI:** 10.1186/s12917-018-1477-1

**Published:** 2018-05-11

**Authors:** Thierry Olivry

**Affiliations:** 1 Department of Clinical Sciences, College of Veterinary Medicine, North Carolina State University, Raleigh, NC 27606 USA; 2 Comparative Medicine Institute, North Carolina State University, Raleigh, NC USA

## Abstract

It has been more than 40 years since the dual descriptions of canine pemphigus vulgaris. Over the ensuing four decades, the reports of—mostly canine—novel autoimmune skin diseases (AISDs) have progressed in successive waves separated by long periods of quiescence. This Editorial introduces a series of comprehensive review papers on the various canine and feline AISDs. This collection of articles aims at remediating the current veterinary literature deficiency on this topic by summarizing the key historical, clinical, histological, immunological and treatment characteristics of animal AISDs.

**Electronic supplementary material:**

The online version of this article (10.1186/s12917-018-1477-1) contains supplementary material, which is available to authorized users.

## Editorial

**Fig. 1:**
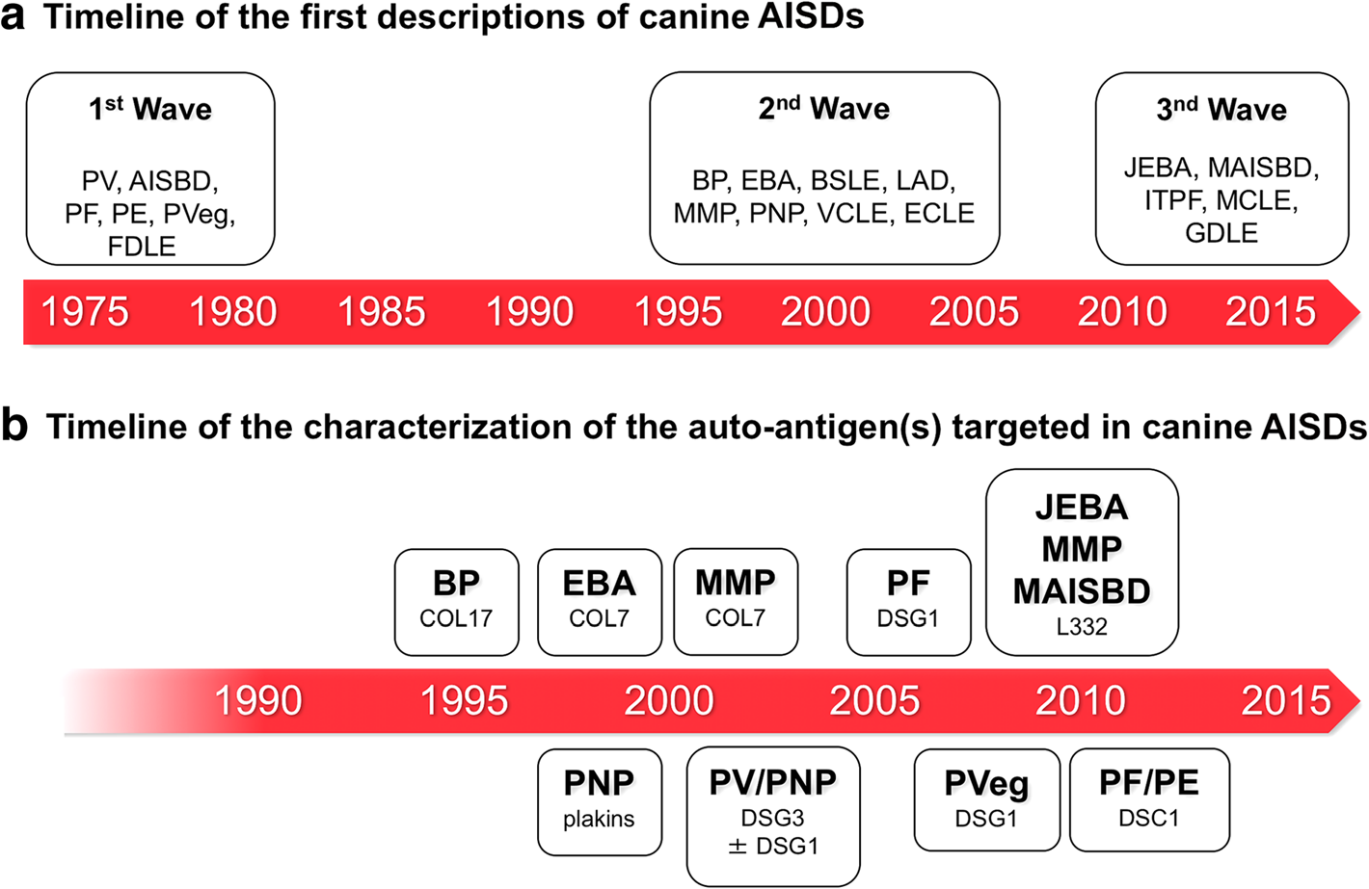
Historical timelines of canine auto-immune skin diseases. **a** Timeline of the first descriptions of canine auto-immune skin diseases. **b** Timeline of the discovery of the targeted auto-antigens in the antibody-mediated auto-immune skin diseases. For abbreviations, please refer to the end of this editorial

It has been more than 40 years since the dual descriptions of canine pemphigus vulgaris [1, 2]. Over the ensuing four decades, the reports of—mostly canine—novel auto-immune skin diseases (AISDs) have progressed in successive waves separated by long periods of quiescence (Fig. 1a). After, or concurrently with these later descriptions, auto-antigen(s) were characterized following their successive discoveries in human AISDs (Fig. 1b).

At first, animal AISDs were separated into “*vesiculous or bullous*” (i.e., the blister-forming pemphigus and pemphigoid variants) and “*non-bullous*” diseases (i.e., discoid and systemic lupus erythematosus) [3]. With the recognition of vesicular cutaneous lupus erythematosus in Collie breeds and the often-non-bullous mucous membrane pemphigoid in dogs and cats, this original classification no longer seemed relevant or of any clinical value. At this time, we propose instead to create a nosology based on the dominant mechanism of lesion formation. This classification additionally provides a simple rationale for the implementation of immunosuppressive treatment regimens designed from the known mechanism of action of the various drugs.

We now suggest to separate the animal AISDs into those with lesions due—or presumed to be due—to the action of auto-antibodies (Additional file 1: Table S1) and those whose lesions are the consequence of the direct attack by—usually cytotoxic—T-lymphocytes (Additional file 2: Table S2). Within these two broad categories of AISDs, entities can be then separated logically along their main cellular or molecular targets.

While there are now many old or more recent single case reports and series of AISDs in dogs, cats, horses—and even goats, cattle, pigs, and sheep—there are no current review papers that summarize the key historical, clinical, histological, immunological and treatment characteristics of animal AISDs.

This new collection of open access articles aims at remediating this veterinary literature deficiency.

At this time, we have planned no less than six reviews on the various canine and feline AISDs. Some will have the typical format of narrative—yet in-depth—reviews (for example this first one on canine cutaneous lupus erythematosus variants), while some others will embrace systematic reviews and meta-analysis principles. Our planned series of the very rare pemphigus vulgaris will take the latter format, which will allow us to gather and regroup all of the information available in every single case report or mechanistic paper ever published on this very rare animal AISD.

Provided that this collection has the success and worldwide diffusion that we hope, we will likely expand it with additional reviews on animal immune-mediated (inflammatory) diseases (IMIDs), which we define as diseases in which a dysregulated immune-response to exogenous—but not self—antigen(s) develops or fails to abate. These diseases are not very well-characterized, and, as will be the case for erythema multiforme, Stevens-Johnson syndrome and toxic epidermal necrolysis, one would need to develop a consensus on clinical characteristics before scrutinizing and summarizing the literature in a usable format.

We hope that this journal’s readership will enjoy this new collection of review articles.

## Additional files

Additional file 1: **Table S1.** Revised classification of auto-antibody-mediated auto-immune skin diseases in animals. Bolded are the most common diseases in the various species. Underlined are major autoantigens, i.e., those recognized by serum auto-antibodies in more than 50% of at least ten patients with the disease. For abbreviations, please refer to the end of this editorial. (DOCX 79 kb)

Additional file 2: **Table S2.** Revised classification of lymphocyte-mediated autoimmune skin diseases in animals. Bolded are the most common diseases in the various species. For abbreviations, please refer to the end of this editorial. (DOCX 45 kb)

## Abbreviations

AA: Alopecia areata
AISD: Auto-immune skin disease
BP: Bullous pemphigoid
BSLE-I: Bullous systemic lupus erythematosus, type I
COL17: Collagen XVII
COL7: Collagen VII
DSC1: Desmocollin-1
DSG1: Desmoglein-1
DSG3: Desmoglein-3
EBA: Epidermolysis bullosa acquisita
ECLE: Exfoliative cutaneous lupus erythematosus
FDLE: Facial discoid lupus erythematosus
GDLE: Generalized discoid lupus erythematosus
IgAP: IgA pemphigus
ITPF: Insecticide-triggered pemphigus foliaceus
JEBA: Junctional epidermolysis bullosa acquisita
L332: Laminin-332
LAD: Linear IgA disease
MAISBD: Mixed auto-immune subepidermal blistering dermatosis
MCLE: Mucocutaneous lupus erythematosus
MMP: Mucous membrane pemphigoid
PE: Pemphigus erythematosus
PF: Pemphigus foliaceus
PG: Pemphigoid of gestation
PNP: Paraneoplastic pemphigus
PP: Pseudopelade
PV: Pemphigus vulgaris
PVeg: Pemphigus vegetans
SA: Sebaceous adenitis
VCLE: Vesicular cutaneous lupus erythematosus
VKH: Vogt-Koyanagi-Harada syndrome
