# Supplementary figures and images for: Auto-immune skin diseases in animals: time to reclassify and review after 40 years
Source: BMC Vet Res. 2018 May 11;14:157. doi: 10.1186/s12917-018-1477-1 (PMC5948701; doi:10.1186/s12917-018-1477-1)

**Table S2: Revised classification of lymphocyte-mediated autoimmune skin diseases in animals**


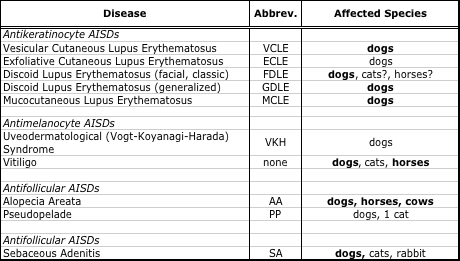

Supplement: Supplementary file 2 — Table S2. Revised classification of lymphocyte-mediated autoimmune skin diseases in animals. Bolded are the most common diseases in the various species. For abbreviations, please refer to the end of this editorial. (DOCX 45 kb) [file 12917_2018_1477_MOESM2_ESM.docx]
